# Self‐Learning Videos in Focused Transthoracic Echocardiography Training

**DOI:** 10.1111/tct.70014

**Published:** 2025-01-02

**Authors:** Diana Morales Castro, Irene Wong, Danny Panisko, Umberin Najeeb, Ghislaine Douflé

**Affiliations:** ^1^ Interdepartmental Division of Critical Care Medicine, Toronto General Hospital University of Toronto Toronto Ontario Canada; ^2^ Department of Medicine University of Toronto Toronto Canada; ^3^ Department of Anesthesia and Pain Management, Toronto General Hospital University Health Network Toronto Ontario Canada

**Keywords:** focused, FOTE, pocus, self‐learning videos, transthoracic echocardiography

## Abstract

**Background:**

Focused transthoracic echocardiography (FOTE) is crucial for patients' bedside management. However, limited opportunities exist for practical FOTE training, prompting the use of simulation and self‐learning videos to overcome this constraint. This study aimed to evaluate the impact of incorporating self‐learning videos into a simulation FOTE training course.

**Approach:**

This was a prospective, randomized study involving University of Toronto internal medicine residents, who participated in a 2‐h didactic and simulation FOTE training course before being randomized to a control group receiving written learning materials or an intervention group with additional self‐directed learning videos.

**Evaluation:**

Twenty‐eight participants were randomized, and twenty‐one (75%) completed the 1‐month follow‐up. Participants were assessed using a written test on image acquisition techniques and structure identification, scanning time and image quality on a simulator and self‐reported scanning comfort, both pre‐intervention and 1‐month post‐intervention. The groups had no significant difference in the time spent reviewing the material (1.5 vs. 1.4 h, *p* = 0.76). A significant increase in post‐course scores was observed in all evaluations except for the control group's written test (*p* = 0.07). There were no significant between‐group differences across the written test (*p* = 0.7), image quality (*p* = 0.6) and comfort level (*p* = 0.7). Compared to the control group, the intervention group exhibited a greater reduction in the scanning time (38 vs. 72 s, *p* = 0.02).

**Implications:**

FOTE training effectively increases theoretical knowledge and practical skills in a simulated setting. However, limited video utilization by participants precluded the inference of definitive conclusions on the impact of self‐learning videos.

## 1 Introduction

The use of focused transthoracic echocardiography (FOTE) to assess critically ill patients with shock and respiratory failure has significantly increased over the last decade [1, 2]. Growing evidence demonstrates that FOTE is a pivotal tool for bedside assessment, diagnosis and management of critically ill patients [3, 4]. Nevertheless, FOTE diagnostic accuracy relies on the clinician's ability to acquire and to analyse the images, making practical training essential to improve proficiency and minimize the risks of diagnostic errors [1, 5, 6, 7].

FOTE is a pivotal tool for bedside assessment, diagnosis and management of critically ill patients.

The scarcity of practical training opportunities presents a major barrier to FOTE adoption, as patient scanning under direct supervision is required [6, 8]. To overcome this limitation, simulators have emerged as an effective tool to teach image acquisition, offering efficiency and reducing dependence on instructors for training [6, 8, 9, 10]. Previous studies have shown that simulator‐based training for anaesthesia and critical care residents leads to improvements in FOTE test scores and image quality [5, 6, 8, 10, 11, 12, 13]. Although self‐learning videos are becoming increasingly available, either online or as part of FOTE training courses, few studies have evaluated their impact on image acquisition quality and theoretical knowledge improvement [1, 14]. This study assessed the impact of incorporating self‐learning videos into a FOTE training course that utilized a high‐fidelity echocardiography simulator (Heartworks). We hypothesize that adding self‐directed learning videos would result in better test scores compared to simulation‐based training alone (Table 1).

**TABLE 1:** Educational methodologies.

International Association for Medical Education (AMEE) curriculum integration framework	Kirkpatrick typology for training evaluation
Plan: Develop a curriculum with expected outcomes Implement: Implement the simulation‐based educational exercises and new curriculum Evaluate: Evaluate effectiveness/assess learning outcomes and satisfaction Revise: Based on results, make revisions to the simulation curriculum	Level 1: Participation: covers learners' views on the learning experience, content, teaching methods, organization, materials and quality of instruction Level 2a: Modification of attitudes and perceptions Level 2b: Modification of knowledge and skills Level 3: Behavioural change, transfer of learning to the workplace Level 4a: Change in organizational practice Level 4b Benefits to patients and clients

## 2 Approach

### 2.1 Study, Setting and Participant Characteristics

This prospective randomized study was conducted from September 2022 to April 2023 with approval from the University of Toronto (UofT) Research Ethics Board (Study number: 31741‐V002) and the Core Internal Medicine Program Director. The first and second year UofT Core Internal Medicine residents were invited to participate via institutional email and recruited on a first‐come, first‐served basis; exclusion criteria included any prior formal echocardiography training. Informed consent was obtained, and the participants could withdraw at any point. Participants were randomized in a 1:1 ratio to either the control group, receiving only the written learning material, or the intervention group, receiving additional self‐directed learning videos. The written material (Supporting Information) provided instructions on obtaining FOTE views, including patient positioning, probe manipulation, scanning sequence, image optimization and echocardiographic anatomy. The self‐learning videos, only accessible to the intervention group, visually demonstrated the manoeuvres and techniques outlined in the written material, including image optimization [How to get the views – Excel (echocardiography.ca)].

## 3 Evaluation

### 3.1 Training Protocol and Evaluation

The participants underwent a three‐part pre‐test. First, a 10 single‐best‐answer question written test evaluating image acquisition techniques and structure identification (Supporting Information). Second, evaluation of scanning abilities consisted in acquiring four standard FOTE views on a simulator (parasternal long axis, parasternal short axis, apical four‐chamber and subcostal views); image acquisition was timed, and each view was graded with a previously validated scale of 1–5 (Table 2) by a National Board of Echocardiography (NBE) certified intensivist, blinded to group allocation [7]. Third, residents rated their scanning comfort on a 10‐point scale.

**TABLE 2:** Assessment scale for image quality.

Grade	Definition
1	Unable to obtain a view or wrong view obtained
2	Minimally recognizable structures. Image quality insufficient for diagnosis
3	Image quality minimally sufficient for diagnosis
4	All structures imaged well. Diagnosis easily supported
5	All structures imaged with excellent image quality. Diagnosis completely supported

They then received a 2‐h in‐person session by an NBE‐certified intensivist following the International Association for Medical Education ‘Guideline for Simulation in Healthcare Education’ curriculum integration framework [8], aimed at Level 2b of the Kirkpatrick methodological approach to training evaluation (Table 2) [4]. This included a didactic lecture reviewing the examination sequence, image acquisition and optimization, recognition of cardiac structures and FOTE limitations. Following this, a review of clinical cases that included patients with no pathology, hypovolemia, left and right ventricular dysfunction, wall motion abnormalities, pericardial effusion and cardiac tamponade, valvular abnormalities and suboptimal echocardiographic views was done. This concluded with a hands‐on session with the simulator.

After 1 month of unlimited access to the learning material and the simulator, the three‐part test was repeated, followed by another clinical case review session. A 6‐month follow‐up session was offered to all participants who completed the 1‐month evaluation.

### 3.2 Measurements and Variables

The primary outcome was the between‐group difference in score change in the pre‐ and post‐course evaluations. An estimate of 22 participants were required to detect a 10% group difference in test scores with 80% power and significance of *p* < 0.05. Twenty‐eight study subjects were recruited to account for possible dropouts and sample size calculation inaccuracy. Secondary outcomes were the score change in each group between the pre‐and post‐course evaluations.

Descriptive statistics using proportions for categorical variables, mean with standard deviation (SD) and median with interquartile ranges (IQR) for continuous variables were used. The Shapiro–Wilk test was used to assess the normality of distribution. Both groups were compared using bivariate analysis. Normally distributed continuous variables were compared using a *t*‐test, and skewed data using the Wilcoxon rank‐sum test. Each group's pre‐ and post‐course scores were compared using the Mann–Whitney *U* test. Between‐group score differences were compared with the two‐way ANOVA. A *p* < 0.05 was considered significant. We used an intention‐to‐treat approach, including the participants' scores that did not complete the course. We also performed a per‐protocol analysis, excluding the subjects who lost follow‐up, and a sensitivity analysis, assigning no change and the average change in scores to the participants who did not complete the post‐course tests, to corroborate the study findings.

## 4 Results

**FIGURE 1:**
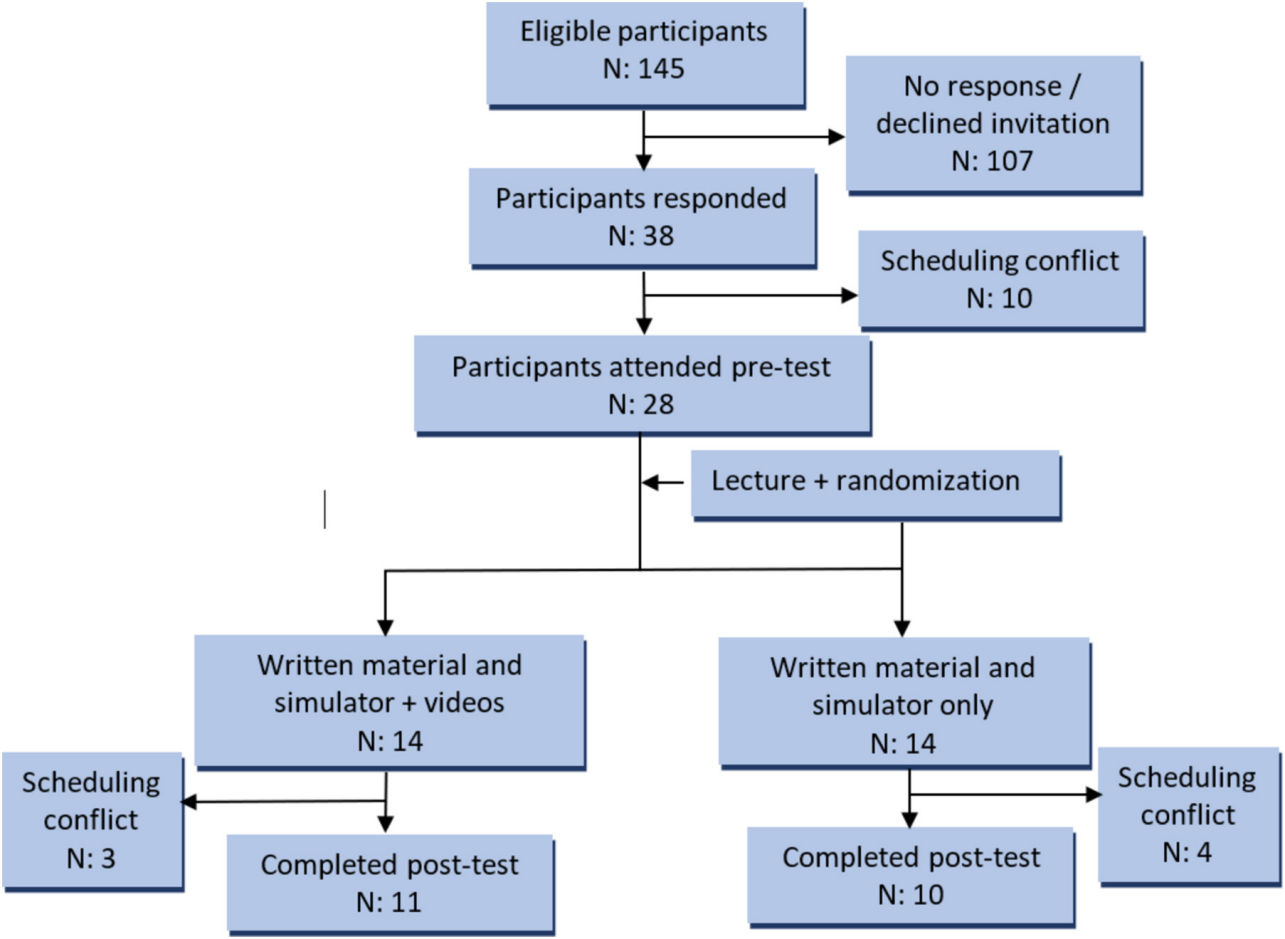
Flow diagram of participants.

A total of 28 participants were randomized and completed the pre‐test and in‐person training. Twenty‐one (75%) participants completed the 1‐month follow‐up session (Figure 1). Three participants attended the 6‐month follow‐up session. Baseline characteristics and pre‐course written test and image quality scores were similar between the two groups (Table 3). The time taken to obtain the four basic views was longer at baseline for the intervention group compared to the control (126 ± 66 vs. 86 ± 71 s, *p* = 0.006).

**TABLE 3:** Participant baseline characteristics and pre‐course test scores.

Characteristic	Group	
	Control (*N* = 10)	Intervention (*N* = 11)
Residency year		
First	9 (90)	6 (56)
Second	1 (10)	5 (44)
Prior scanning experience	3 (27)	3 (31)
Number of previous scans	0 (0–1.5)	0 (0–0)
Written test^a^	48 ± 20	43 ± 24
Image quality^a^	44 ± 19	34 ± 17
Scanning time, seconds	86 ± 71	126 ± 66
Comfort level^a^	27 ± 19	20 ± 16

*Note:* Data are expressed in number (percentage), mean ± standard deviation or median (IQR).

^a^ Percentage grade.

During the period between the pre‐ and post‐test, there were no significant between‐group differences in the time spent reviewing the material (1.5 ± 0.8 vs. 1.4 ± 0.8 h, *p* = 0.76), the time scanning on the simulator (1.1 ± 0.8 vs 1.5 ± 1.0 h, *p* = 0.47) or the number of actual patients scanned [median (IQR) = 1 [1–3] vs. 1 [1, 2], *p* = 0.37]. In the intervention group, the average time spent reviewing videos was 1.1 ± 0.7 h, two participants did not review the videos, and two participants reviewed extra materials not provided in the course. In the control group, one participant did not review the written material, and two participants reviewed videos from another source.

**FIGURE 2:**
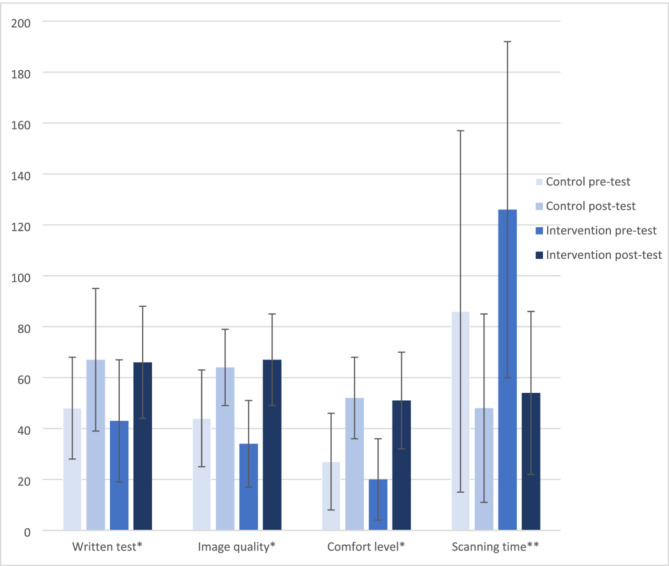
Pre‐ and post‐test scores for both control and intervention groups separated by the test components.

Both groups showed significant improvements across all the evaluated components, except for the written score in the control group (Figure 2). However, when comparing the two groups, the absolute improvements in written test scores (intervention 23% vs. control 19%, *p* = 0.7), image quality (intervention 33% vs. 20%, *p* = 0.6) and self‐reported comfort level (intervention 31% vs. 25%, p = 0.7) were not significantly different. The only significant difference was a greater reduction in the scanning time in the intervention group compared to control group (72 s vs. 38 s, *p* = 0.02).

### 4.1 Per‐Protocol and Sensitivity Analysis

Using per‐protocol analysis, we consistently found a significant increase in scores for all the components in both groups (Table S1). However, there remained no significant improvement in written test scores and image quality in the intervention group compared to the control. Consistent findings resulted from the sensitivity analysis, both with the lowest and highest scores assigned (Table S2).

### 4.2 Implications

#### 4.2.1 Important Results and Relevance

In this study, a 2‐h didactic FOTE course with a hands‐on learning session using a high‐fidelity echocardiography simulator resulted in a Level 2b Kirkpatrick improvement in FOTE knowledge and practical skills, aligning with prior research on simulation effectiveness in FOTE teaching [3, 4, 5, 6]. The overall low pre‐test scores (< 50%) confirm the participants' lack of experience prior to participating in the course. The large score increase in both groups confirmed that formal training significantly increased participant's skills and knowledge [6]. The incorporation of self‐learning videos did not significantly improve test scores, although it did reduce scanning time. The notable improvement in the control group may have masked the potential benefits of adding videos. Additionally, the intervention group's longer pre‐test scanning time may explain their greater reduction in scanning time. Although extrapolation of results to other fields of sonography, such as gynecology or abdominal sonography, is limited due to differing learning curves. Our findings contribute to the growing body of evidence supporting the value of simulator training in FOTE education.

The incorporation of self‐learning videos did not significantly improve test scores, although it did reduce scanning time.

#### 4.2.2 Strengths and Limitations

Our study had several strengths. The outcome assessor was blinded to training group allocation, eliminating grading bias. Also, the score increase was consistent in both, the per‐protocol and sensitivity analysis. However, the study had multiple limitations. First, this was a single‐centre study conducted in a simulated setting with a small sample size, which was limited by the number of residents interested in participating in this voluntary study. This voluntary nature may have introduced selection bias towards residents interested in FOTE, and the small sample size limits the generalizability of our findings, as the results might not represent the broader resident population. Moreover, the lack of statistically significant differences could be attributed to insufficient power. It is possible that with a larger cohort, the observed improvements in scores from the self‐learning videos could have reached statistical significance. Second, all participants, except one, reviewed the self‐learning materials for less than 2 h. We could not ensure that the intervention group reviewed all the videos or ascertain that they reviewed them more than once. Third, there was a significant drop‐out rate further affecting the study power. The course was non‐mandatory, requiring participation during residents' free time, with no obligation to complete the training. Residency training obligations took priority if schedules conflicted. Finally, the low 6‐month follow‐up precluded the evaluation of longitudinal retention, which was variable in previous studies [5, 6]. The translation of acquired skills into clinical practice needs to be further tested with studies involving real‐life patient imaging to evaluate the curriculum impact at higher Kirkpatrick levels [9].

## 5 Conclusion

FOTE training consisting of didactic teaching, clinical case review and the use of a high‐fidelity echocardiography simulator effectively increased theoretical knowledge, practical skills in a simulated setting and scanning comfort of trainees. Adding self‐learning videos did not provide additional improvement. However, more insight into participants' low usage of the self‐directed learning materials is required. Further studies evaluating learners in a clinical setting are also necessary.

More insight into participants' low usage of the self‐directed learning materials is required.

## Author Contributions

D.M.C., I.W., D.P., U.N. and G.D. designed and planned the study. D.M.C. and I.W. collected, analysed and interpreted the data. D.M.C. wrote the first draft. All authors were involved in the critical revision of the final manuscript and approved it.

## Ethics Statement

The study has been approved by the University of Toronto Health Sciences ethics board, approval number 31741‐V002.

## Conflicts of Interest

The authors declare no conflicts of interest.

## Supporting information


**Figure S1** Transducer movements.
**Figure S2.** Parasternal long‐axis view.
**Figure S3.** Parasternal short‐axis ‐apillary level.
**Figure S4.** Apical four chamber.
**Figure S5.** Subcostal four chamber.
**Table S1.** Per protocol analysis of pre‐ and post‐course test scores.
**Table S2.** Sensitivity analysis for difference in pre‐and post‐test scores.

## Data Availability

The data that support the findings of this study are available from the corresponding author upon reasonable request.

## References

[1] J. L. Diaz‐Gomez , S. Perez‐Protto , J. Hargrave , et al., “Impact of a Focused Transthoracic Echocardiography Training Course for Rescue Applications Among Anesthesiology and Critical Care Medicine Practitioners: A Prospective Study,” Journal of Cardiothoracic and Vascular Anesthesia 29, no. 3 (2015): 576–581. 25622973 10.1053/j.jvca.2014.10.013

[2] R. M. Mazraeshahi , J. C. Farmer , and D. T. Porembka , “A Suggested Curriculum in Echocardiography for Critical Care Physicians,” Critical Care Medicine 35, no. 8 Suppl (2007): S431–S433. 17667468 10.1097/01.CCM.0000270280.65365.AA

[3] J. Gaudet , J. Waechter , K. McLaughlin , et al., “Focused Critical Care Echocardiography: Development and Evaluation of an Image Acquisition Assessment Tool,” Critical Care Medicine 44, no. 6 (2016): e329–e335. 26825858 10.1097/CCM.0000000000001620

[4] M. Biswas , R. Patel , C. German , et al., “Simulation‐Based Training in Echocardiography,” Echocardiography 33, no. 10 (2016): 1581–1588. 27587344 10.1111/echo.13352

[5] N. T. Townsend , J. Kendall , C. Barnett , and T. Robinson , “An Effective Curriculum for Focused Assessment Diagnostic Echocardiography: Establishing the Learning Curve in Surgical Residents,” Journal of Surgical Education 73, no. 2 (2016): 190–196. 26774938 10.1016/j.jsurg.2015.10.009

[6] J. Neelankavil , K. Howard‐Quijano , T. C. Hsieh , et al., “Transthoracic Echocardiography Simulation Is an Efficient Method to Train Anesthesiologists in Basic Transthoracic Echocardiography Skills,” Anesthesia and Analgesia 115, no. 5 (2012): 1042–1051. 22822190 10.1213/ANE.0b013e318265408f

[7] G. Douflé , R. Teijeiro‐Paradis , D. Morales‐Castro , et al., “Point‐of‐Care Ultrasound: A Case Series of Potential Pitfalls,” Case 6, no. 6 (2022): 284–292. 36036054 10.1016/j.case.2022.05.002 PMC9399537

[8] D. Canty , J. Barth , Y. Yang , et al., “Comparison of Learning Outcomes for Teaching Focused Cardiac Ultrasound to Physicians: A Supervised Human Model Course Versus an eLearning Guided Self‐ Directed Simulator Course,” Journal of Critical Care 49 (2019): 38–44. 30359924 10.1016/j.jcrc.2018.10.006

[9] K. C. See , J. W. Chua , D. Verstegen , J. J. G. Van Merrienboer , and W. N. van Mook , “Focused echocardiography: Dyad versus individual training in an authentic clinical context,” Journal of Critical Care 49 (2019): 50–55. 30366250 10.1016/j.jcrc.2018.10.009

[10] C. A. Rambarat , J. M. Merritt , H. F. Norton , E. Black , and D. E. Winchester , “Using Simulation to Teach Echocardiography: A Systematic Review,” Simulation in Healthcare 13, no. 6 (2018): 413–419. 30520805 10.1097/SIH.0000000000000351

[11] R. Morgan , B. Sanville , S. Bathula , S. Demirel , R. S. Perkins , and G. E. Johnson , “Simulator‐Based Training in FoCUS With Skill‐Based Metrics for Feedback: An Efficacy Study,” Pocus 4, no. 2 (2019): 33–36.

[12] T. Edrich , R. R. Seethala , B. A. Olenchock , et al., “Providing Initial Transthoracic Echocardiography Training for Anesthesiologists: Simulator Training Is Not Inferior to Live Training,” Journal of Cardiothoracic and Vascular Anesthesia 28, no. 1 (2014): 49–53. 24183827 10.1053/j.jvca.2013.07.011

[13] P. Vignon , B. Pegot , F. Dalmay , et al., “Acceleration of the Learning Curve for Mastering Basic Critical Care Echocardiography Using Computerized Simulation,” Intensive Care Medicine 44, no. 7 (2018): 1097–1105. 29931488 10.1007/s00134-018-5248-z

[14] J. Heiberg , L. S. Hansen , K. Wemmelund , et al., “Point‐of‐Care Clinical Ultrasound for Medical Students,” Ultrasound International Open 1, no. 2 (2015): E58–E66. 27689155 10.1055/s-0035-1565173 PMC5023212

