# Supplementary material for: Self‐Learning Videos in Focused Transthoracic Echocardiography Training
Source: Clin Teach. 2025 Jan 2;22(1):e70014. doi: 10.1111/tct.70014 (PMC11695197; doi:10.1111/tct.70014)
Supplement: Supplementary file 1 — Figure S1 Transducer movements. Figure S2. Parasternal long‐axis view. Figure S3. Parasternal short‐axis ‐apillary level. Figure S4. Apical four chamber. Figure S5. Subcostal four chamber. Table S1. Per protocol analysis of pre‐ and post‐course test scores. Table S2. Sensitivity analysis for difference in pre‐and post‐test scores. [file TCT-22-e70014-s001.docx]

**Supplemental Material**

*Learning material*

FOTE tutorial series

**The 2D TTE examination sequence**

The purpose of material is to standardize the performance of FOTE studies and to help the learner optimize and troubleshoot image acquisition.

Patient positioning

Even in the intensive care unit, you should try to position the patient in left lateral decubitus for apical and parasternal windows, unless there is a contraindication, such as spine precautions or when inappropriate in case of severe cardiovascular or respiratory instability. The goal is to shift the heart laterally. Often, positioning a wedge or a pillow underneath the right side is necessary to support this position. If possible, raise the patient’s left arm above the head to increase the size of the intercostal spaces.

Your Position

Try to practice performing the examination from either side of the patient; in ICU one side may not be easily accessible due to presence of other equipments. However, unless you are performing an emergency study, try to position yourself in a comfortable position. Make sure that the ultrasound system is located close to you and the patient, to allow simultaneous scanning and manipulation of the settings.

The first step requires recording of patient identification data (e.g., full name, date of birth and identification number) and essential clinical data (vital signs, height and weight, ventilator settings and vasoactive agents, if applicable). If the patient is on any form of extra-corporeal life support, this information should also be recorded. Another crucial step is the placement and connection of the ECG electrodes. Make sure you have a good ECG signal on your monitor. Always remember to save the images acquired.

Sequence

The sequence starts from the parasternal long axis views, you will then move to the parasternal short axis at the level of the papillary muscles. After completing the parasternal views, move to the apical four chamber view, from this view you can obtain the apical 5-chamber view. For the subcostal windows, obtain the subcostal 4-chamber view followed by view of the inferior vena cava.

Handling the probe

For adult transthoracic echocardiography performed by cardiologists and intensivists, two main rules are applied:

1. The top of the screen is closest to the probe, with deeper structures displayed at the bottom of the screen
2. The probe marker corresponds to the right side of the screen, as indicated by a marker on the top right of the ultrasound sector.

It is paramount that you verify your orientation prior to beginning any exam. For example, emergency physicians often use a different and opposite convention, with the screen marker on the left top corner of the ultrasound sector.

Another essential component of the ultrasound examination is the correct transducer manipulation. According to the American Institute of Ultrasound In Medicine (AIUM), we can identify five primary movements of the transducer: 1) sliding; 2) rotating; 3) tilting; 4) rocking; 5) compression. To correctly describe and understand these movements, we will refer to the following key components of an ultrasound transducer: marker, main axis, footprint (shape of the transducer) and tail or posterior part of the probe (Figure 1):

1. Sliding: The first movement is the sliding, which refers to the overall movement of the probe towards a specific direction or area of the body.
2. Rotating: The transducer can also be rotated around the axis of the transducer footprint, in a clockwise or counterclockwise direction. The relative position of the probe indicator provides spatial orientation for this type of movement. For example, when the marker is directed cephalad, it is considered facing 12 o’clock. A 90 degree clockwise rotation of the transducer, will determine a turn of the marker from 12 o’clock to 3 o’clock.
3. Rocking: Rocking is the movement of the probe along its main axis, changing only the angle of the insonation of the transducer in this axis while maintaining a fixed point on the body. Rocking the transducer toward or away from the marker will allow you to center the area of interest or to extend the field of view. This movement is also called “in plane” motion.
4. Tilting: In contrast, tilting refers to a movement of the probe in a plane perpendicular to its main axis along a fixed point on the body while changing the angle of insonation. Tilting is therefore a motion that is perpendicular to rocking. Tilting the transducer allows to visualize different planes in the same axis without sliding the transducer on the body. For example, the tilting motion allows visualization from the base to the apex of the heart in the parasternal short axis view or of the IVC and aorta in the subcostal longitudinal view.
5. Compression is defined as pressure applied on the transducer, with the transducer footprint kept at a fixed position. It can be used to make adequate contact between the transducer footprint and the patient’s body, thus improving image quality. Always remember to minimize as much as possible the compression to maintain the patient’s comfort.

Figure 1. Transducer Movements.

**The views**

**Parasternal long axis view**

Obtaining the view

The parasternal long axis view can be obtained by placing the probe between the 2^nd^ and 4^th^ intercostal space along the left parasternal border (close but not over the sternum). The transducer indicator should be pointing toward the patient’s right shoulder.

Always start setting the depth at 20-24 cm; this will allow to adequately assess

1) possible presence of left pleural effusion and pericardial effusion

2) descending thoracic aortic pathology.

Subsequently, you will decrease the depth to focus on cardiac structures (usually the deapth will be set between 10 and 16 cm).

The criteria for an optimal parasternal long axis view are the following:

- the apex of the left ventricle should not be visualized
- the mitral valve should be positioned in the middle of the screen, with the aortic valve slightly positioned on the right
- the interventricular septum and the infero-lateral wall should appear horizontal
- 2 cusps of the aortic valve should be clearly visualized and appear symmetrical

Optimization

Careful manipulation of the transducer will allow you to optimize the view in the vast majority of patients.

If the apex of the left ventricle is visualized on the screen, two manipulations should be attempted:

1. Rocking of the probe toward the sternum
2. Sliding of the probe toward the sternum

If the aortic cusps do not appear symmetric, a slight rotation of the transducer should be performed in either direction. If rotating the transducer is insufficient to optimize visualization aortic cusps, tilting the tail of the probe in either direction may help.

Similarly, you should be able to insonate the entire LV long axis in its maximum diameter. To achieve this goal, two transducer manipulations can be attempted:

1. Rotating the transducer in either direction will allow you to identify a sonographic plane that is parallel to the left ventricular long axis
2. You may then tilt the tail of the transducer upward or downward.

You may have to adjust both rotation and tilting several times until you obtain an adequate view

Finally, if the interventricular septum and the infero-lateral wall do not appear horizontal , 2 manipulations can be attempted:

1. Sliding of the transducer superiorly (usually one intercostal space higher)
2. Rocking the footprint toward the sternum

Figure 2. Parasternal long axis view.

**Parasternal short axis view- Papillary muscles level**

Obtaining the view

To obtain the parasternal short axis views, from the parasternal long axis view, you will have to rotate the transducer clockwise between 60 and 90 degrees. The transducer marker should be directed toward the left shoulder. This may result in an over rotation and the marker may need to be redirected between the sternal notch and the mid clavicular-line.

When starting from the parasternal long axis view, if the mitral valve is visualized on the center of the screen, you are most likely to obtain the parasternal short axis view at the level of the mitral valve upon clockwise rotation of the probe. Similarly, if the aortic valve is visualized on the center of the screen in the parasternal long axis view, you are most likely to obtain the parasternal short axis view at the level of the aortic valve. Once you have obtained one of the parasternal views (mitral valve or aortic valve level), you should tilt the transducer tail toward the right shoulder for the papillary muscles view.

Optimization

The left ventricle should appear circular in shape. Sometimes, the left ventricle will instead appear more oval and oblong (“pear-shaped”). This could be due to 2 acquisition mistakes:

- 1. Over-clockwise rotation;
  2. Position of the transducer excessively low.

Therefore, every time the left ventricle does not appear as a circular structure, try to rotate the transducer counterclockwise. If the rotation does not improve your view, if the interventricular septum appears flat instead of curved or if you see the tricuspid valve, you may try sliding the transducer one intercostal space higher.

Lastly, if the left ventricle is not in the center of your view, you will have to slide or rock towards or away from the left shoulder. If the left ventricle appears on the right half of the screen, slide or rock the transducer towards the left shoulder. If the left ventricle appears on the left half of the screen, slide or rock it away from the left shoulder.

Remember to slightly move on the skin to optimise your resolution.

Figure 3. Parasternal short axis- Papillary level

**Apical four-chamber view**

Obtain the view

The apical four-chamber view is usually found near the point of maximum impulse. Most of the time, the transducer will be positioned between the 4^th^ and 6^th^ intercostal space between the mid-clavicular and mid-axillary lines. In the vast majority of patients this point will be below and lateral to the left nipple.

The location of the optimal acoustic window varies significantly with the patient’s position; for example in supine patients the optimal apical window will be significantly more medial.

The transducer marker should be pointing toward the patient’s left, with a slight counterclockwise rotation, approximately between 2 and 3 o’clock.

You should direct the ultrasound beam in the direction of the sternal notch

In the ideal apical 4-chamber view,

- the septum appears perfectly vertical;
- the left ventricular apex is positioned in the center of the scanning sector;
- all 4 chambers (right and left atria, right and left ventricle) are visible and coaptation planes of tricuspid and mitral planes are both visible
- the left ventricle is not foreshortened.

Optimization

1. The apex not being centered is one of the most common problems. If the left ventricular apex is positioned towards the right side of the screen and you see mostly the right ventricle, you will have to slide the transducer more laterally. Conversely, if the left ventricular apex is positioned towards the left side of the screen, you will have to slide the transducer more medially.
2. Often the septum will appear oblique instead of vertical. More frequently, the septum will appear directed from the bottom left of the screen to the top right of the screen. In that case, you will have to either direct the transducer tail more laterally. If instead the septum is directed from the bottom right of the screen to the top left of the screen, tilt the tail of the probe more medially.
3. If you visualize the coronary sinus and the left atrium is cut-off, you are directing the ultrasound beam too posteriorly. You will therefore have to tilt the tranducer tail downward, to direct the ultrasound beam more anteriorly.
4. On the other hand, if you visualize the aortic valve, your are directing the ultrasound beam too anteriorly. You will therefore have to tilt the tranducer tail upward, to direct the ultrasound beam more posteriorly.
5. In case of poor definition of the ventricular walls, a slight counterclockwise rotation from the 3 o’clock position may help you get a better visualization of the later walls as the transducer will better fit in the intercostal space.
6. Lastly, you should ensure you are imaging the true left ventricular apex and not foreshortening the left ventricle. The true apex can be identified by its thin wall and its relative lack of motion. If the ventricle looks foreshortened and more “globular” than bullet-shaped, try to slide the transducer one or more lower intercostal spaces until you visualize the true apex.

Figure 4. Apical four chamber

**Subcostal Views**

Patient Position

**To obtain the subcostal view you will position the patient supine. And to facilitate your movement you should hold the probe from above as if you were holding a screw driver.**

Subcostal four-chamber

**Obtaining the view**

**Start from the subxyphoid process, from the right side of the abdomen to use the liver as an acoustic window. If the patient can cooperate you may ask the patient to bend her or his knees.**

**For the subcostal 4 chamber, the marker should be oriented towards the patient’s left at roughly 2 o’clock. The probe itself should be almost flat on the patient, and try to get the footprint closer to the subxyphoid process.**

**Optimization**

**If the aortic valve is seen, it means that you are looking too anteriorly. To correct this, you will have to lift the tail up to look more posteriorly. This may not be sufficient and you may have to also rotate slightly counterclockwise.**

**To bring the apex more in the middle, bring the tail towards the right of the patient. To bring the right atrium in the middle bring the tail towards the left of the patient.**

**Figure 5. Subcostal four chamber**

**Subcostal IVC**

**Obtaining the view**

**To obtain the Inferior vena cava, from the subcostal 4 chamber view, bring first the right atrium in the middle and then rotate counterclockwise keeping the right atrium in the center. You should aim to rotate between 60 and 90 degrees, the marker should be looking towards the patient’s head. Fine rotational movement should be applied to make sure that the long axis of the inferior vena cava is imaged.**

**The optimal view of the IVC should allow you to visualize the IVC RA junction, the liver on both sides, the sus hepatic vein draining in the IVC and the IVC should be open as far as possible into the liver.**

**Optimization**

**Sometimes a slight tilting in either direction may also help. You should be able to see the IVC RA junction and the sus hepatic vein. If you cannot see the IVC RA junction try to slide cephalad on the abdomen or rock the probe cephalad.**

**If when rotating you image the descending aorta, just tilt the footprint towards the patient’s right which should enable you to get the IVC.**

**You can also obtain the IVC view starting from the short axis of the IVC by placing the probe directly flat on the patient’s abdomen with the marker directed to the left at 3 o’clock. You can rotate on the axis of the IVC counterclockwise to obtain the long axis of the IVC with the marker ultimately positioned upwards.**

Make sure that you are not imaging the sus hepatic vein that can have an insertion in the IVC very close to the IVC RA junction. They can be recognised as they are more oblique than the IVC, go into one of the lobes of the liver and also have multiple ramifications.

*Theory test*

Correct answers are highlighted with bold.

1. Which structure do you want to assess when you perform the parasternal long axis view with 20-24 cm depth?

**a. descending aorta**

b. inferior vena cava

c. right pleural space

d. right ventricle

2. Which of the following structures will not be visualized on the parasternal short-axis papillary level?

- - 1. **pulmonary artery**
    2. right ventricle
    3. left ventricle
    4. papillary muscles

3. How will you obtain the apical five-chamber from an apical four-chamber?

a. rotate counter-clockwise

b. tilt posteriorly (probe tail up)

c. slide anteriorly (towards the sternum)

**d. tilt anteriorly (probe tail down)**

4. When performing an inferior vena cava subcostal view, the transducer marker is facing:

a. to the left shoulder

b. to the right shoulder

**c. to the head of the patient**

d. to 9’ o clock

5. Which of the following is characteristic of an appropriate apical four-chamber view?

a. foreshortened ventricles

**b. vertical interventricular septum**

c. right ventricle in the center of the screen

d. visualizing the aortic valve

6. To obtain a parasternal short-axis view:

a. the marker should be towards the right shoulder of the patient

b. from the apical four-chamber rotate 45 degrees counter-clockwise

c. from the parasternal long axis rotate 45 degrees counter-clockwise

**d. the marker should be towards the left shoulder of the patient**

7. Which of the following structures will not be visualized on the apical four-chamber?

a. tricuspid valve

**b. pulmonic valve**

c. mitral valve

d. moderator band

8. Regarding the short axis at the papillary muscle level:

a. the right ventricle cannot be seen

b. only the anterolateral papillary muscle is appreciated

**c. is ideal to assess for wall motion abnormalities**

d. the left ventricle appears with a crescent shape

9. If the aortic valve is seen in the subcostal four-chamber, you should

**a. lift the tail of the transducer up**

b. rotate clockwise 90 degrees

c. ask the patient to turn towards the left side

d. rock (along the transducer main axis) to the left

10. You receive a patient after major intra-abdominal surgery. The patient is hypotensive (BP 85/40) and tachycardic (HR 130) with a lactate of 7 despite fluid resuscitation. You perform a FOTE, and you see a moderate pericardial effusion, in this case:

a. there is no cardiac tamponade if there is no chamber collapse

b. if the IVC is not plethoric cardiac tamponade can be ruled out

**c. cardiac tamponade is a clinical diagnosis**

d. FOTE is not the appropriate image technique for cardiac tamponade

Supplemental Table 1. Per protocol analysis of pre-and post-course test scores

| **Groups** | **Control** | **Intervention** | ***p*-value for between group comparison** |
| --- | --- | --- | --- |
| **Written test*** |  |  |  |
| Pre-test | 42 ± 24 | 41 ± 25 | 0.9 |
| Post-test | 65 ± 25 | 69 ± 23 |  |
| Difference | 23 (*p*= 0.03) | 28 (*p*= 0.03) | 0.8 |
| **Image Quality*** |  |  |  |
| Pre-test | 43 ± 21 | 37 ± 18 | 0.6 |
| Post-test | 64 ± 14 | 68 ± 19 |  |
| Difference | 21 (*p*= 0.02) | 31 (*p*= 0.007) | 0.9 |
| **Comfort level*** |  |  |  |
| Pre-test | 21 ± 18 | 26 ± 19 | 0.6 |
| Post-test | 53 ± 15 | 50 ± 25 |  |
| Difference | 32 (*p*= 0.001) | 27 (*p*= 0.03) | 1.0 |
| **Scanning time**** |  |  |  |
| Pre-test (min) | 97 + 77 | 122 + 69 | 0.1 |
| Post-test (min) | 53 + 48 | 51 + 43 |  |
| Difference (min) | 44 (*p*= 0.009) | 71 (*p*< 0.001) | 0.8 |

*Score in percentage, ** time in seconds

Note: the difference row represents the comparison between the absolute differences between the

pre- and post-intervention scores/

Supplemental Table 2. Sensitivity analysis for difference in pre-and post-test scores

| **Group** | **Score change** | ***p*-value** |
| --- | --- | --- |
| **Control** |  |  |
| **No change** |  |  |
| Written test | 15.7 ± 26.5 | 0.06 |
| Image quality | 10.0 ± 13.7 | 0.01 |
| Comfort level | 20.0 ± 19.6 | 0.01 |
| **Average change** |  |  |
| Written test | 21.1 ± 24.5 | 0.02 |
| Image quality | 18.6 ± 10.4 | < 0.001 |
| Comfort level | 27.2 ± 14.6 | 0.001 |
| **Intervention** |  |  |
| **No change** |  |  |
| Written test | 22.1 ± 27.8 | 0.02 |
| Image quality | 25.7 ± 17.6 | 0.004 |
| Comfort level | 19.3 ± 19.4 | 0.009 |
| **Average change** |  |  |
| Written test | 27.1 ± 25.1 | 0.005 |
| Image quality | 32.7 ± 10.8 | 0.001 |
| Comfort level | 28.0 ± 14.8 | 0.002 |
